# White Matter Injury After Intracerebral Hemorrhage

**DOI:** 10.3389/fneur.2021.562090

**Published:** 2021-06-10

**Authors:** Xiongjie Fu, Guoyang Zhou, Jianfeng Zhuang, Chaoran Xu, Hang Zhou, Yucong Peng, Yang Cao, Hanhai Zeng, Jianru Li, Feng Yan, Lin Wang, Gao Chen

**Affiliations:** Department of Neurosurgery, Second Affiliated Hospital, School of Medicine, Zhejiang University, Hangzhou, China

**Keywords:** spontaneous intracerebral hemorrhage, white matter injury, demyelination, axonal damage, stroke

## Abstract

Spontaneous intracerebral hemorrhage (ICH) accounts for 15% of all stroke cases. ICH is a devastating form of stroke associated with high morbidity, mortality, and disability. Preclinical studies have explored the mechanisms of neuronal death and gray matter damage after ICH. However, few studies have examined the development of white matter injury (WMI) following ICH. Research on WMI indicates that its pathophysiological presentation involves axonal damage, demyelination, and mature oligodendrocyte loss. However, the detailed relationship and mechanism between WMI and ICH remain unclear. Studies of other acute brain insults have indicated that WMI is strongly correlated with cognitive deficits, neurological deficits, and depression. The degree of WMI determines the short- and long-term prognosis of patients with ICH. This review demonstrates the structure and functions of the white matter in the healthy brain and discusses the pathophysiological mechanism of WMI following ICH. Our review reveals that the development of WMI after ICH is complex; therefore, comprehensive treatment is essential. Understanding the relationship between WMI and other brain cells may reveal therapeutic targets for the treatment of ICH.

## Introduction

Spontaneous intracerebral hemorrhage (ICH) is a serious disease and a global public healthcare challenge. ICH accounts for 10–15% of all stroke cases in Western countries and 20–30% of those in Asia (1). ICH is associated with high morbidity and mortality. Up to 30% of patients die within 30 days (2). Approximately 20% of survivors with major neurologic deficits regain their ability to perform daily living functions independently after 6 months (3). The disease places a heavy burden on countries and families (4). The most important independent risk factors for ICH are age and hypertension. Its incidence increases with age, and 70–80% of the patients with ICH are found to be hypertensive (4–8). ICH is associated with other comorbidities, including diabetes mellitus, coronary heart disease, and amyloid angiopathy (5, 8).

ICH develops when ruptured blood vessels cause extravasation of blood into the brain parenchyma. Brain injury after ICH is usually primary and secondary. Primary brain injury occurs in the hyper-acute stage of ICH and involves mechanical damage to the perihematoma tissue. The degree of primary injury is dependent on the location and volume of the hematoma (9, 10). The predilection location is in the basal ganglia, thalamus, and internal capsule, which are rich in white matter fibers and are easily damaged by the mechanical stress of a hematoma (11, 12). Secondary brain injury after ICH is a complex process that includes processes such as neuroinflammation, oxidative stress, iron deposition, brain edema, and damage to the blood–brain barrier (BBB) (9, 13).

In the past, many basic studies of ICH have focused on neuronal death, gray matter damage, and neuroinflammation. However, few effective treatments are available in clinical practice (9, 13). Brain injury following ICH is a complicated pathophysiological process involving many cells and structures. The white matter may present another therapeutic target. Currently, multiple studies are focused on white matter injury (WMI). They discuss its mechanism of injury and the relationship between WMI and ICH outcomes (14–18). Although clinical and basic studies are increasing, information on the mechanisms of WMI and its effective treatment remains limited.

Previous studies of ICH have shown that rodents are the appropriate experimental animal models. They accurately mimic the natural process of WMI after ICH in humans (19). The proportion of white matter in the rodent brain is 10–20%; however, it is 50% in humans (11, 20). This indicates that WMI plays a vital role after ICH; thus, it is important to understand it in detail. Past studies have reviewed WMI after ICH, although not systematically or comprehensively (11, 19, 21). Therefore, this review summarizes the latest findings on the development of WMI after ICH in terms of anatomical structure, function, mechanism of injury, potential treatment, and repair of WMI after ICH to improve the outcome of ICH patients.

## Method

In this review, we conducted a systematic online search in PubMed (https://pubmed.ncbi.nlm.nih.gov), without time limits, for papers published in English, appearing with the following keywords: WMI, white matter lesion, white matter hyperintensities, white matter change, leukoaraiosis or leukoencephalopathy, ICH, intracerebral hemorrhage, spontaneous intracerebral hemorrhage, or cerebral hemorrhage. References appearing in the reference lists of the articles provided by the online search were also used.

## The Anatomical Structure and Function of White Matter

The healthy adult brain consists of gray and white matter. The white matter occupies ~50% of the brain's volume (22) and consists of myelinated and unmyelinated axons, fiber tracts, and supporting glial cells, whereas the gray matter consists of neuronal cell bodies, glial cells, and blood vessels (23). The white matter is primarily located deep within the brain and includes the subcortical and periventricular regions, where there is usually poor blood supply (24, 25). Myelin sheaths are produced by mature oligodendrocytes and cover the long axons (11). Myelin provides electrical insulation and allows signals to pass quickly and accurately between the brain regions (21). Myelin sheaths degrade, causing loss of axonal integrity, which affects the accuracy and efficiency of neural signal transduction (11). The function of the white matter in the central nervous system (CNS) includes supporting and connecting the gray matter in various brain regions, regulating the distribution of action potentials, and acting as relay stations and coordinators of neuron signal transmission (26). It also serves as the intersection of the efferent and afferent nerve fibers for information exchange between the central and peripheral nervous systems (27, 28). The location and characterization of the white matter make it more vulnerable to insult by factors such as mechanical stress, hypoxia, and neuroinflammation.

White matter fibers can be divided into three types based on their distribution and connections (29). Projection fibers transmit signals from the cortex to other brain locations such as the corticospinal tracts, spinocerebellar tracts, optical radiations, and thalamocortical radiations. Commissural tracts link the left and right cerebral hemispheres, which include the corpus callosum. The transverse distribution of white matter fibers connects one cortical lobe with another within the ipsilateral hemisphere such as the longitudinal and the uncinate fasciculus (11, 21).

Apart from signal relay and coordinating communication, white matter has been thought to be involved in learning and cognitive processes (30). After injury, patients can have cognitive dysfunction, sensorimotor impairment, psychiatric symptoms, gait disturbances, urinary incontinence, pain, and other neurological deficits (31). In addition, the normal aging process is accompanied by a decrease in the length of white matter fibers, which is related to cognitive impairment (32, 33). WMI is different from gray matter injury, as it may be reversible with appropriate treatment within a limited time window. Consequently, certain brain functions can be restored (34).

## General Condition of White Matter Injury After Intracerebral Hemorrhage

In experimental rat ICH models, the blood is distributed along white matter tracts during bleeding, causing atrophy and injury (35). Previous studies have primarily focused on neuronal and gray matter injury. With further research, WMI after ICH has gained attention (35). The majorly affected locations are the perihematoma region and corpus callosum, whereas the anterior and hippocampal commissure are less affected (36). Demyelination and axonal damage are the primary manifestations of WMI. In experimental rat models, demyelination occurred in the core and at the edge of the hematoma, which began 1 day after ICH and peaked at 3 days. Axonal damage was observed at the edge of the hematoma as early as 6 h following ICH. It peaked at 3 days, and then decreased for up to 28 days (14, 37). Demyelination and axonal damage were not observed at 28 days after ICH either in the core or the perihematoma (38). The degree of axonal damage was more serious in aging rats; however, there was no difference in demyelination between the young and older rats (37). Although there is significant and severe WMI in the acute phase of ICH, this injury continues into the chronic phase in the natural course of the disease (39).

Mature oligodendrocytes are key white matter components, especially in subcortical white matter glial cells, where they occupy ~75% of the volume (40). The mature oligodendrocytes in the hematoma and perihematoma regions die after ICH due to the effects of multiple injurious factors (41). Immature oligodendrocyte precursor cells (OPCs) infiltrated the perihematoma region, the number increases dramatically at 3 and 7 days after ICH, and then decreases to normal levels at 28 days (41). OPCs infiltrated and differentiated into mature oligodendrocytes, which leads to myelin regeneration. The number of mature oligodendrocytes increases dramatically as measured at 7 and 14 days and then decreases and reaches normal levels at 28 days (15).

## Mechanisms of White Matter Injury After Intracerebral Hemorrhage

**Figure 1:**
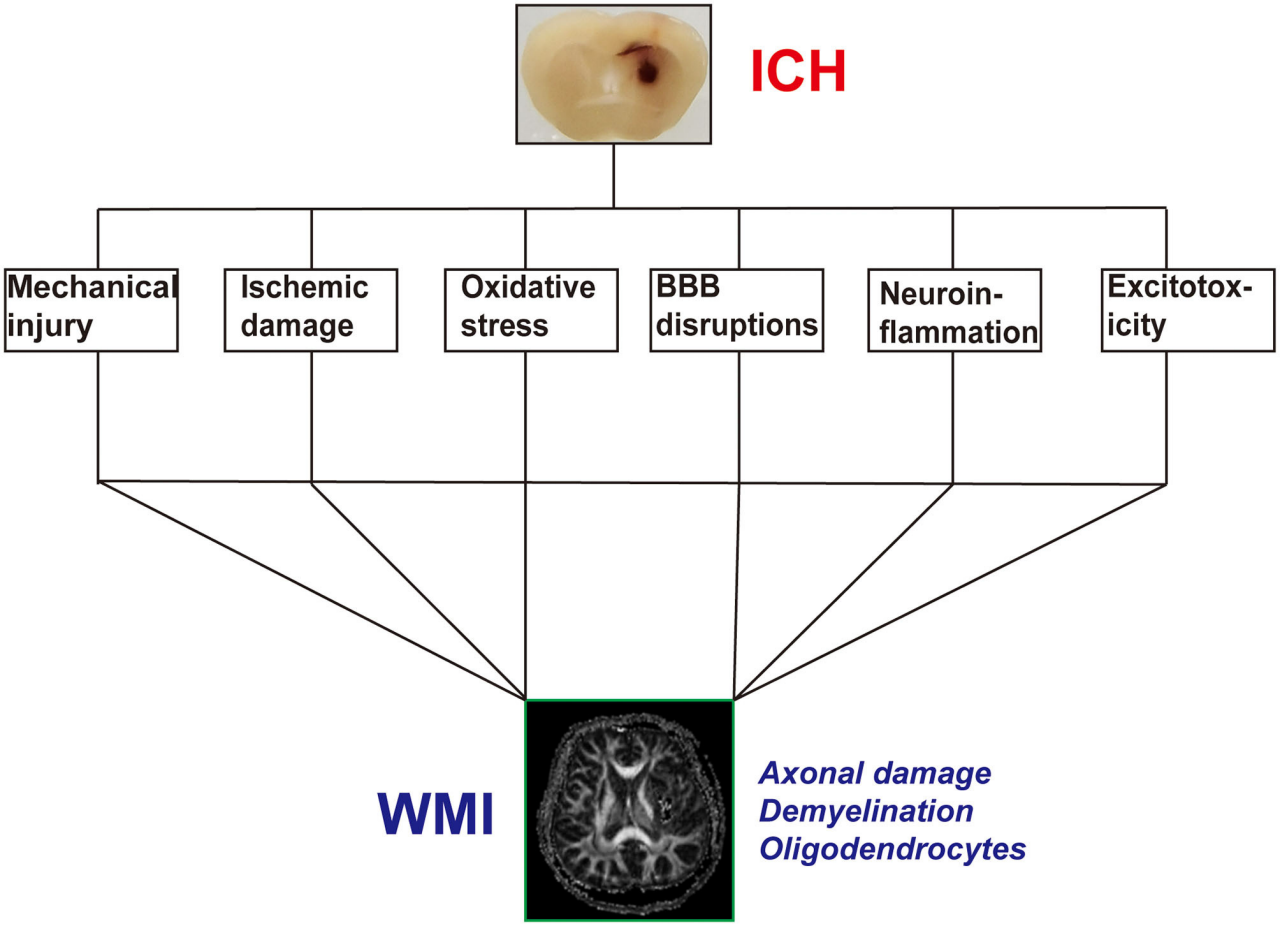
The possible pathophysiological factors of white matter injury (WMI) after intracerebral hemorrhage (ICH).

The development of WMI after ICH is a complex process involving multiple factors, regions, and mechanisms. However, the detailed underlying mechanisms remain unclear. We have examined previous reports on the mechanisms of WMI following ICH (Figure 1).

### Mechanical Injury

Mechanical injury occurs during the hyper-acute stage and lasts for an extended period of time. White matter fibers are more vulnerable than other cells in the CNS to mechanical injury because of their distribution and shape. After ICH, the blood extravasates into the brain parenchyma and forms a hematoma. The location and volume of hematoma are major factors associated with the degree of WMI (10). This action directly damages white matter fibers in the hematoma core. These fibers have difficulty healing. Secondary mechanical injury is induced by the edema and massive effect of the hematoma, which compresses the fibers in the perihematoma region. The degree of WMI depends on the velocity of blood and the hematoma size (21). Secondary mechanical injury of the white matter can be relieved by surgical removal and manipulation of the mechanical receptors over time. In WMI of ICH origin, force from the fluid percussion waves causes sudden expansion of the hematoma. However, in traumatic brain injury (TBI), the force results from an external blow to the head (42).

### Ischemic Damage and Oxidative Stress

The hematoma mass effect and stimulation of the perihematoma parenchyma causes severe edema. Such edema occurs promptly, lasts longer, reduces cerebral blood flow, and causes white matter ischemic damage (43). The normal healthy brain accounts for ~20% of the whole body's blood supply, has almost no excess oxygen or glucose reserves, and suffers devastating consequences when the oxygen supply is disrupted (44). Furthermore, the white matter is usually located deep inside the brain, where there is poor blood supply. The structures of the white matter, including the axons and oligodendrocytes, are more sensitive to ischemic injury than those of the gray matter (45).

Oxidative stress is a critical injurious factor that leads to WMI. After ICH, brain injury is usually accompanied by an increase in reactive oxygen species (ROS) and other oxidative stress responses (46). After several days, erythrocytes lyse and release a series of metabolites such as hemoglobin, heme, iron, and bilirubin, which generate abundant ROS and WMI (47, 48). The major source of ROS is iron overload after ICH. Intra-striatal injection of FeCl_3_ causes distinct demyelination and axonal damage. Iron chelator treatments can decrease iron deposition and attenuate WMI (38). Other erythrocyte lysis products, such as bilirubin, also cause WMI through ROS (49). An increase in ROS causes direct axonal and oligodendrocyte damage (50). Upregulated ROS also interacts with injury factors, thereby aggravating BBB disruption and neuroinflammation (51, 52).

### Blood–Brain Barrier Disruptions

Previous studies have shown that the BBB destruction is common in WMI after acute brain injury (53, 54). Maintenance of BBB homeostasis is dependent on the glia–vascular unit, which consists of white matter, astrocytes, oligodendrocytes, endothelial cells, and microglia (55, 56). After BBB disruption, vascular permeability increases and results in toxic blood components to entering the brain parenchyma, which can exacerbate brain edema, axonal damage, demyelination, and oligodendrocyte death (57). Furthermore, due to congenital factors, the BBB structure is incomplete in some special white matter regions. Therefore, the white matter becomes easily damaged after brain injury (58). After ICH, activation of matrix enzymes such as matrix metalloproteinase (MMP)-9 can damage the BBB and degrade the vascular basement and tight junction proteins (59). BBB disruption can cause peripheral blood-derived immune cells such as neutrophils to infiltrate the brain tissue, and positive feedback worsens BBB damage and then exacerbates WMI (14, 59).

### Neuroinflammation

Apart from the fibers, the white matter also contains glial cells and oligodendrocytes, astrocytes, and microglia. After brain damage, many pro-inflammatory glial cells are activated and secrete cytokines and chemokines (60), which can further activate glial cells. This forms a vicious cycle. Previous studies have indicated that acute brain injury, such as TBI and ischemic and subarachnoid hemorrhage, results in the activation of pro-inflammatory cells and the release of pro-inflammatory cytokines, which are closely related to WMI. Treatment with anti-inflammatory agents can relieve WMI and improve neural function (46, 61, 62). Microglia, the major component of pro-inflammatory glial cells, have two phenotypes: pro-inflammatory (M1) and anti-inflammatory (M2). M1 microglia secrete pro-inflammatory cytokines that can aggravate WMI, damage oligodendrocytes, and cause neurological deficits. In addition, M2 microglia can secrete brain-derived neurotropic factors, which increase the recovery of the damaged white matter. Therefore, interventions that shift M1 to M2 can reduce WMI and improve neurological function (61, 63, 64). Although the phenotype of microglia has been extensively researched, the view of the microglia phenotype is that of simplicity, which cannot adequately describe the complex physiology of microglial cells (65). In future studies, newly developed technologies should be applied to explore microglial signatures (66). Astrocytes are another important cellular component of ICH-induced neuroinflammation. The phenotype of astrocytes also includes two types: A1 astrocytes (pro-inflammatory) and A2 astrocytes (anti-inflammatory). A1 astrocytes can upregulate and express pro-inflammatory cytokines and chemokines, which may exacerbate the neuroinflammatory response and WMI (66, 67). A2 astrocytes can encourage the upregulation of neurotrophic factors and the secretion of proteins that promote recovery (68).

Neuroinflammation plays a key pathophysiological role in ICH. After ICH, damage-associated molecular patterns released by many brain structures and cells activate local glial and peripheral immunocyte infiltration cells and cause severe neuroinflammatory responses (69, 70). Although ICH can lead to marked neuroinflammatory responses, the relationship between neuroinflammation and WMI is unclear. Lakovic et al. (49) showed that the products of hematoma lysis activate microglia and astrocyte and release multiple pro-inflammatory cytokines that cause direct oligodendrocyte apoptosis and exacerbate the degree of WMI. The c-Jun N-terminal kinase (JNK) pathway mediates inflammation after ICH (71). When the JNK pathway is activated, it leads to the production of pro-inflammatory cytokines, which can induce demyelination, axonal damage, and oligodendrocyte apoptosis (39, 72). In conclusion, neuroinflammation is an important injurious factor in WMI after ICH.

### Excitotoxicity of Glutamate

Previous research has shown that the level of glutamate increases and correlates with the degree of brain edema and BBB disruption (73). In addition, the glutamate level at the perihematoma is associated with the outcome of ICH patients post-surgery (74). The predilection location of ICH is the basal ganglia, thalamus, and internal capsule, which are rich in white matter fibers (11, 12). Postoperative ICH patients with lower levels of glutamate in the perihematoma have a better prognosis (74), although the effect of glutamate after ICH is not well-understood. Other acute damage to the CNS, such as TBI, cerebral ischemia, and spinal cord injury, causes an increase in glutamate and activates corresponding receptors in the oligodendrocytes and axons with Ca^2+^ overload. This leads to demyelination, axonal damage, and oligodendrocyte death (75–77). Therefore, the glutamate may be involved in WMI after ICH.

## The Evidence of White Matter Injury After Intracerebral Hemorrhage

Immunohistochemical staining is a common method used to detect WMI in experimental animal (rat, mouse, and pig) ICH models (blood and collagenase). The common biomarkers are myelin basic protein (MBP) for normal myelin, degraded myelin basic protein (dMBP) for demyelination, amyloid precursor protein (APP), and neurofilament heavy polypeptide (SMI32) for axonal damage. After ICH, the level of MBP decreases and the levels of APP, dMBP, and SMI32 increase (14, 37, 38, 78, 79). Lively and Schlichter (80) found that the anti-adhesive matricellular protein SC1 is a novel marker of WMI and is highly sensitive to WMI compared to other markers. Luxol fast blue, another tissue stain, can identify normal myelin in lesion regions and can be used to detect WMI at the last stage of ICH (39). Brain tissue histopathological studies show that WMI presents as spongiform changes and tissue rarefaction, with widening perivascular spaces, and reduced brain tissue density (81). Oligodendrocytes are key components for producing myelin and remyelinating axons. After ICH in mice, they die and OPCs are induced and proliferate and differentiate into mature oligodendrocytes. Olig2 is a molecular marker of oligodendrocyte-lineage cells, which are upregulated and persist throughout the life of oligodendrocyte-lineage cells. Hence, it cannot be used as a marker to identify the different states of oligodendrocytes (82–84). The common molecular markers are Olig2/NG2 for OPCs and Olig2/CC1 for mature oligodendrocytes (15, 85).

The neurofilament light (NFL) chain is a marker for neuroaxonal damage in many CNS diseases, including ischemic stroke, subarachnoid hemorrhage (SAH), TBI, and ICH (86–89). After ischemic stroke, the level of serum NFL can reflect the degree of damage to the white matter and predict the short- and long-term clinical outcomes (89, 90). In the early phase of SAH, NFL is significantly elevated in the cerebrospinal fluid (CSF) and serum. The concentration of NFL in the plasma is also a strong predictive biomarker for the clinical outcome of SAH 30 days after SAH onset during the early brain injury phase (87, 91). In patients with mild TBI, NFL levels can reflect chronic axonal degeneration and dysregulation (86). In patients with ICH, the level of plasma NFL is highly elevated and correlated with the hematoma volume. The level of NFL in plasma may be a promising biomarker for outcome prediction in ICH (88). Although the NFL level reflects the amount of axonal damage in many CNS diseases, the predictive value of plasma NFL in ICH patients with white matter damage requires further research. Based on the potential clinical value and its strong ability to predict axonal injury in nervous system disease, it is reasonable to suggest that NFL can be a biomarker candidate for tissue damage in the pathophysiology process of WMI after ICH.

Transmission electron microscopy (TEM) is a tool used to evaluate the WMI. Previous research found that the morphological changes in the ultrastructure of white matter in the ICH mouse model were remarkably similar to those in multiple sclerosis (16). In acute ICH, the number of myelinated axons is decreased. The remaining axons have larger diameters but thinner myelin sheaths, and the space between the axons is increased and filled with debris (49). Zhao et al. (92) used TEM to evaluate the ultrastructure of the myelinated nerve fibers and found that, after ICH, the myelin sheath became loose and swollen, and the layers were disorganized, exhibiting an onion-like appearance. In the ICH mouse model, the ipsilateral striatum and corpus callosum of hematoma axons showed an obvious pattern of demyelination and loss of uniformity in size and shape. Furthermore, the axonal bundles were fragmented and myelin appeared fractured (93).

**Figure 2:**
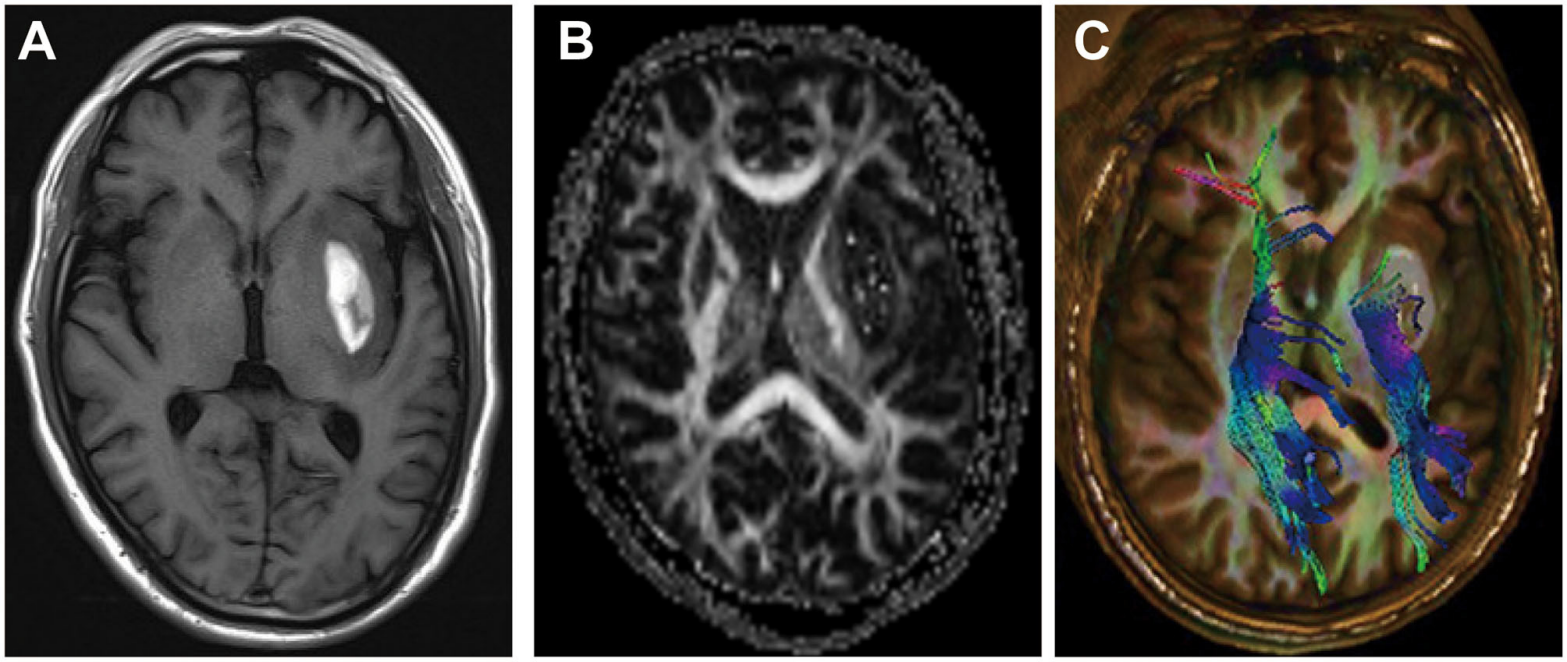
Example of white matter injury (WMI) after intracerebral hemorrhage (ICH). **(A)** T1-weighted image after ICH. **(B,C)** Diffusion tensor images of WMI after ICH.

Magnetic resonance imaging (MRI) is a non-invasive imaging technique that allows three-dimensional (3D) assessment of brain tissue and has been widely used to evaluate the structure and function of the white matter (94) (Figures 2A–C). WMI after ICH presents as hyperintensities on T2 sequences, which correlate with the hematoma volume (46, 81, 95). However, T1 and T2 sequences cannot accurately assess the WMI. Diffusion tensor imaging (DTI) can detect the thermal movement of water molecules in white matter with a higher sensitivity than conventional MRI sequences in WMI. By measuring parameters such as fractional anisotropy, mean diffusivity, axial diffusivity, and radial diffusivity, DTI can evaluate the integrity and connectivity of fibers, rebuild the 3D distribution of white matter pathways in injured regions, and help determine the microstructural pathophysiology (21). After ICH, DTI revealed that WMI presents as decreased fractional anisotropy, axial diffusivity, and increased radial diffusivity (96).

## Treatment of White Matter Injury After Intracerebral Hemorrhage

### Removal of the Hematoma

As mentioned above, the hematoma can directly damage white matter fibers in the hematoma core and compress the fibers in the perihematoma region. Surgical removal may be an effective treatment for ICH, as it can relieve nerve compression and the toxic stimulation of the perihematoma region, lower intracranial pressure, and improve patient outcomes. The main surgical methods include craniotomic hematoma dissection, stereotactic hematoma removal, and the endoscopic hematoma evacuation (97). However, the optimal method remains unknown. With advances in micro-neurosurgery, minimally invasive hematoma evacuation is a common surgical technique used to treat ICH. The results of three small randomized trials showed that minimally invasive surgery with instillation of urokinase can improve functional outcomes; however, it has no effect on reducing the risk of death (97–99). The results of the MISTIE III trial showed that stereotactic hematoma removal did not improve functional outcomes compared with standard medical care in patients with large hematomas (100).

### Inhibition of Oxidative Stress

Oxidative stress is an important injurious factor that leads to WMI, and it can be relieved. After ICH, treatment with zinc protoporphyrin (ZnPP) inhibited the degradation of hemoglobin and alleviated WMI (101). Baicalein can increase superoxide dismutase and glutathione peroxidase activities and downregulate malondialdehyde, thereby alleviating oxidative stress (102). Administration of isoliquiritigenin in the acute ICH phase relieves neurological deficits *via* regulation of ROS/nuclear factor (NF)-κB on the activation of the NLRP3 inflammatory pathway by triggering Nrf2 activity and the Nrf2-induced antioxidant system (102). HC-030031, an antagonist of transient receptor potential ankyrin 1, can relieve WMI by inhibiting ROS production (16). Mitoquinone, a selective mitochondrial ROS scavenger, reduces demyelination and axon swelling by blocking ATP depletion and mitochondrial ROS overproduction (16). The deposition of iron increases ROS production. Thus, iron chelators can decrease ROS levels and alleviate WMI (103).

### Reducing Neuroinflammation

Neuroinflammation is a key pathological mechanism of WMI after ICH; therefore, reducing inflammatory reactions can alleviate WMI. Neutrophils are an important component of neuroinflammation. Using corresponding antibodies to deplete circulating blood neutrophils can reduce activated microglia/macrophages and decrease demyelination and axon damage (14, 104). Microglia are a major component of pro-inflammatory glial cells and have two proposed phenotypes. VK28 and IL33 treatment drives M1 into the M2 phenotype, which can ameliorate WMI and improve neurological function (78, 105). Iron deposition after ICH causes inflammatory cell activation and the release of inflammatory cytokines, which induce WMI and neurological dysfunction. Iron chelators such as 2,2′-dipyridyl and deferoxamine can suppress the inflammatory response through the tumor necrosis factor (TNF)-α/receptor-interacting protein kinase 1 (RIPK1) and JNK pathways (39, 103, 106). Treatment with FYT720, an immune-modulating drug, can regulate the polarization of microglia and decrease the level of TNF-α, thereby improving WMI outcomes (106). In a study by Yang et al. (107), the results showed that inhibition of histone deacetylases promoted microglia toward the M2 (anti-inflammatory) phenotype and alleviated WMI and the neurological outcomes induced by ICH, which may involve the JAK/signal transducer and activator of transcription (STAT) signaling pathway.

Minocycline, a tetracycline antibiotic, can reduce microglial activation and alleviate WMI in pigs through the transforming growth factor (TGF)-β/mitogen-activated protein kinase (MAPK) pathway (17). Minocycline is also able to suppress iron deposition and induce WMI and c-JNK activation after ICH in rats (38, 107). FPS-ZM1, a receptor for advanced glycation end-product (RAGE)-specific antagonist, decreases WMI after ICH (108). SC51089, a prostaglandin E2 type 1 receptor (EP1R) antagonist, decreases Src kinase phosphorylation and MMP-9 activity and relieves axonal damage (109). Supplementing taurine, a CNS amino acid, can reduce neuroinflammation and improve the outcome of WMI (92). Autophagy of microglia can promote neuroinflammation; therefore, reducing inflammation may delay WMI development (110). Another study found that New Interacting Motif E shot (NIMoEsh) could inhibit the activity of JNK and thus improve inflammation and WMI after ICH in mice (72).

## Remyelination and Axon Regeneration

After ICH, many factors induce demyelination and are associated with mature oligodendrocyte death. OPCs migrate to the lesion and differentiate into mature forms, which are key factors for remyelination. *In vitro*, insulin-like growth factor-1 (IGF-1) and platelet-derived growth factor (PDGF) increase OPC proliferation (110–112), and microglia regulate the differentiation process of oligodendrocytes. M1 microglia eliminate damaged cells and degrade myelin sheaths, thereby facilitating remyelination. M2 microglia secrete neurotrophic factors such as brain-derived neurotrophic factor (BDNF), which assists in remyelination (113). Rosiglitazone, a peroxisome proliferator-activated receptor-γ agonist, increases M2 microglia, reduces M1 microglia, and induces OPC differentiation (114). Lithium, as an inorganic salt, has been safely used for the treatment of bipolar disorder in the clinical setting. Li et al. (115) showed that lithium treatment might exert therapeutic efficacy on WMI after ICH through endogenous BDNF signaling in mice, resulting in remyelination.

Axonal dysfunction causes severe symptoms; therefore, its regeneration is a potential treatment for WMI. A study showed that hydrogels help to regenerate axons after peripheral nerve injury, and this may also be beneficial in ICH recovery (116). After CNS injury in adults, modulation of melanopsis/G protein-coupled receptor (GPCR) signaling promotes axon regeneration, which may be exploited in ICH (117). Recently, exosomes have attracted attention because they show great promise as therapeutics for certain diseases. Treatment of rats with mesenchymal stem cell-derived exosomes after ICH maintained the integrity of white matter fibers, stimulated axonal sprouting, and initiated white matter repair (45, 118).

Other treatments include stem cell transplantation, gene therapy, and molecular therapy, although the mechanisms of action of these therapies are not fully understood. Therefore, additional research is required to develop novel treatments for WMI after ICH.

## Limitations

**Figure 3:**
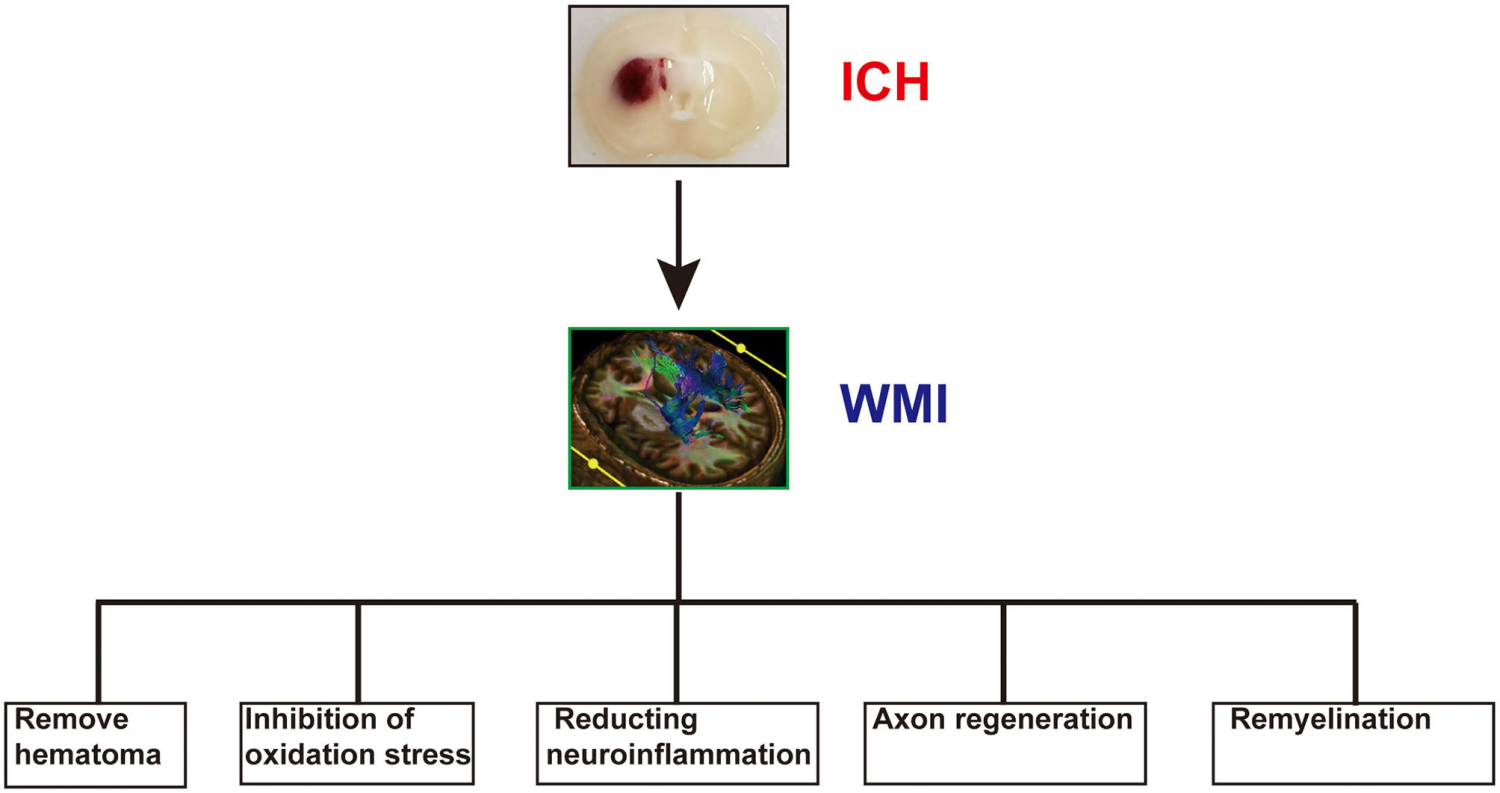
The potential therapeutic strategy of white matter injury (WMI) after intracerebral hemorrhage (ICH).

Although our work provides a comprehensive review of WMI after ICH, our study has several limitations. First, in our review, we focused only on WMI of ICH. However, WMI is a common complication of many CNS diseases, such as ischemic stroke, TBI, and Alzheimer's disease. Further studies are required to obtain additional details concerning other acute and chronic neurological diseases related to WMI. Second, we explored the treatment of WMI after ICH (Table 1 and Figure 3); however, the optimal treatment remains unknown. Therefore, further studies are necessary to determine the ideal treatment for WMI after ICH. Third, many studies on WMI after ICH have been conducted in animal models, so the clinical transformation value of our conclusion is limited. Therefore, further studies are essential to explore the mechanism of WMI in ICH.

**Table 1:** The treatment of WMI after ICH.

**References**	**Model**	**Main results**
Teernstra et al. (119)	Randomized controlled clinical trial	Stereotactic aspiration can be performed safely and in a relatively uniform manner, and improve outcome
Wang et al. (98)	Randomized controlled clinical trial	The minimally invasive craniopuncture technique can improve the independent survival of small basal ganglion patient
Moxon Emre et al. (14)	Collagenase injection in rats	Neutrophil depletion reduced axon loss and neuroinflammation after ICH
Wu et al. (103)	Autologous blood injection in old mice	Pretreatment with lipid soluble iron chelator decreased the accumulation of iron in perihematoma, neuronal death, and WMI
Xie et al. (106)	Autologous blood injection in pigs	Dexferoxamine reduced WMI, TNF-α, and receptor interacting protein kinase 1 levels after ICH in piglets
Ni et al. (39)	Autologous blood injection in rats	Deferoxamine can reduce ICH-induced JNK activation and WMI
Yang et al. (108)	Autologous blood injection in rats	The inhibitor of receptor for advanced glycation end-products can effectively prevent WMI and brain edema after ICH
Zhao et al. (109)	Collagenase injection in mice	EP1R inhibition reduced oxidative stress, WMI, and brain atrophy after ICH
Gu et al. (101)	Autologous blood injection in rats	ZnPP can attenuate ICH-induced WMI
Zou et al. (38)	Autologous blood and FeCl_3_ injection in rats	Minocycline inhibited demyelination and axonal damage in perihematomal tissue after ICH
Wei et al. (102)	Collagenase injection in rats	Baicalein has a proposed anti-inflammatory, antioxidative, and anti-apoptosis effect and may be a novel drug for ICH treatment
Zeng et al. (120)	Collagenase injection in rats	Isoliquiritigenin attenuated the brain injury after ICH involved in the regulation of ROS and NF-κB on the activation of NLRP3 inflammasome pathway by the triggering of Nrf2 activity and Nrf2-induced antioxidant system
Li et al. (105)	Collagenase injection and autologous blood in middle-aged, aged male mice and young female mice	VK-28 polarized microglia to an M2 phenotype, reduced brain water content, decreased WMI, improved neurobehavioral performance, and reduced overall death rate after ICH
Xia et al. (16)	Autologous blood injection in mice	Inhibition of TRPA1 in the acute phase of ICH could ease the perihematoma WMI
Yang et al. (17)	Autologous blood injection in pigs	Minocycline attenuates WMI after ICH through TGF-β-mediated signaling
Zhang et al. (72)	Collagenase injection in mice	NIMoEsh inhibited the neuroinflammation, WMI, and neuronal death after ICH through JNK signaling pathway
Chen et al. (78)	Autologous blood injection in rats	IL-33 promoted microglia M2 polarization and reduced neuronal damage and WMI after ICH
Hanley et al. (100)	Randomized controlled clinical trial	Stereotactic hematoma removal did not improve functional outcome compared with standard medical care in patients with large hematoma
Yang et al. (121)	Collagenase injection in mice	FTY720 treatment could reduce WMI, neuron loss, and neuroinflammation after ICH
Chen et al. (18)	Autologous blood injection in mice	MitoQ can attenuate WMI and improve outcome after ICH through inhibition of mitochondrial injury after ICH
Yang et al. (107)	Autologous blood injection in rats	Inhibiting HDACs promoted microglia toward the M2 phenotype and alleviated WMI and neurological outcome induced by ICH through the JAK/STAT signaling pathway
Yang et al. (122)	Autologous blood injection in mini-pigs	Quantitative susceptibility mapping (QSM) is a non-invasive and reliable method for the assessment of iron-mediated brain injury. Minocycline could reduce brain iron overload, brain edema, and WMI after ICH
Li et al. (115)	Autologous blood injection in mice	Lithium treatment might mitigate WMI after ICH through endogenous BDNF signaling

*BDNF, brain-derived neurotrophic factor; CH, intracerebral hemorrhage; EP1R, prostaglandin E2 type 1 receptor; HDAC, histone deacetylase; JNK, c-Jun N-terminal kinase; NIMoEsh, New Interacting Motif E shot; STAT, signal transducer and activator of transcription; TGF, transforming growth factor; TNF, tumor necrosis factor; WMI, white matter injury; ZnPP, zinc protoporphyrin; ROS, reactive oxygen species; NLRP3, NOD-like receptor pyrin domain-containing protein 3; TRPA1, transient receptor potential cation channel, member A1*.

## Perspective

Although many studies have focused on WMI after ICH, the underlying mechanism is not yet fully understood. Therefore, effective treatment strategies for WMI after ICH are lacking. Further research should focus on exploring an effective therapeutic strategy for WMI after ICH. The proportion of white matter in the human brain is 50%, whereas, in ICH patients, WMI targeted a more severe outcome. Imaging technology can provide a detailed assessment of WMI in patients with ICH. Therefore, investigating new and novel imaging technologies provides an exciting direction for further research.

## Conclusion

A series of pathophysiological changes causes both gray and white matter injuries after ICH. Most studies have focused on gray matter injury, while both gray and white matter are equally important in maintaining normal brain function. Therefore, the present treatments are not comprehensive. With increased investigation, WMI-based treatments may provide novel directions for basic research on ICH. Developing treatments that target both white matter and gray matter injuries may alleviate brain damage and improve patient' outcomes after ICH.

## Author Contributions

GC conceived and designed the paper. XF wrote the paper. GZ and JL assisted in writing the paper. LW collected the data. FY designed how to collect data from online sources. YP developed the original plan. YC hints and advises. JZ coordinated data collection. HZh proofread. CX participated in the discussion. HZe contributed analysis tools. All authors contributed to the article and approved the submitted version.

## Conflict of Interest

The authors declare that the research was conducted in the absence of any commercial or financial relationships that could be construed as a potential conflict of interest.
